# Prognosis and value of preoperative radiotherapy in locally advanced rectal signet-ring cell carcinoma

**DOI:** 10.1038/srep45334

**Published:** 2017-03-27

**Authors:** Chun-Run Ling, Rui Wang, Mo-Jin Wang, Jie Ping, Wen Zhuang

**Affiliations:** 1 Department of Gastrointestinal Surgery, West China Hospital, Sichuan University, Chengdu 610041, China; 2 Department of Gastroenterology, West China Hospital, Sichuan University, Chengdu 610041, China; 3 Center for Quantitative Sciences, Vanderbilt University School of Medicine, Nashville, TN 37232, USA

## Abstract

As well known, signet-ring cell carcinoma (SRCC) is a rare histological subtype of colorectal adenocarcinoma, which has been associated with poor prognosis and resistant to non-surgery therapy compared with common adenocarcinoma. In this study, we assessed the effect of preoperative radiotherapy (PRT) for locally advanced rectal SRCC in a large patient group from the Surveillance, Epidemiology, and End Results program (SEER, 1988–2011) database. SRCC was found in 0.9% (n = 622) rectal cancer (RC) patients in our study. In the PRT setting, SRCC had significantly worse cancer-specific survival than mucinous adenocarcinoma and nonmucinous adenocarcinoma patients (log-rank, *P* < 0.001). In terms of SRCC, stage III RC patients benefited from PRT (log-rank, *P* < 0.001) while stage II did not (*P* = 0.095). The multivariate Cox proportional hazard model showed that PRT was an independent benefit factor in stage III rectal SRCC patients (HR, 0.611; 95% CI, 0.407–0.919; *P* = 0.018). In conclusion, SRCC was an independent predictor of poor prognosis in stage III RC patients, but not in stage II. In the PRT setting of locally advanced RC, SRCC patients had significantly worse prognosis. PRT was an independent prognostic factor associated with improved survival in stage III rectal SRCC.

As a major health burden worldwide, colorectal cancer (CRC) remains the second leading cause of cancer related mortality in the United States^1^,^2^. While adenocarcinomas are the most common tumours of the colon and rectum, variant histological subtypes have been reported to be associated with varied clinical feature and survival. As a rare histology subtype of adenocarcinoma, signet-ring cell carcinoma (SRCC) is defined by the World Health Organization (WHO) containing abundant intracellular mucin in more than 50% of its cells^3^,^4^,^5^. SRCC is found in 0.1–2.6% of CRC patients^6^,^7^. However, there are obvious regional differences. In some countries, such as Jordan and Lebanon, the frequency of SRCC is reported to reach 18.5%^8^. On the other hand, the frequency is only 1% in United States^9^. SRCC is recognized to constitute a distinct pathological entity within the spectrum of CRCs. There are several consistent findings focused on differences among SRCC, mucinous adenocarcinoma (MC) and nonmucinous adenocarcinoma (NMC) in the colorectum. They have demonstrated that SRCC was related to younger age at diagnosis, more advanced stage at presentation and a poorer survival compared with MC and NMC^4^,^5^,^10^,^11^. SRCC presented extensive lymphatic spread, more frequently with multiple metastatic sites and high risk of peritoneal metastases^12^. Some studies have reported molecular and genetic differences among SRCC, MC and NMC, contributing to a more aggressive biological behaviour^13^.

The treatment of rectal cancer (RC) patients has improved rapidly in recent years. Many oncologists considered RC patients with stage II–III as an indication for preoperative radiotherapy (PRT), and recommend to add chemotherapy for patients with locally advanced RC in which the mesorectal fascia is threatened^4^,^14^,^15^. The efficacy of neo-adjuvant therapy including PRT for RC has been described in many literatures^16^,^17^,^18^. SRCC was prone to extensive lymphatic spread and peritoneal metastases leading to poor survival. However, the PRT concerning rectal SRCC patients, which is limited to a low incidence, has been rarely reported^17^,^19^.

In this study, we analysed the clinicopathological characteristics of SRCC and established the prognostic implication of SRCC on locally advanced RC patients who were treated with PRT. Then we determined whether or not locally advanced rectal SRCC patients benefit from PRT.

## Results

### Patient characteristics

A total of 69,543 RC patients were included in this study and most patients were diagnosed with NMC (n = 63,036, 90.6%). MC (n = 5,885) and SRCC (n = 622) were found in 8.5% and 0.9% of patients, respectively (Table 1). The SRCC group presented a younger diagnosis age (59.4 years) than that of NMC (65.4 years) and the MC (65.4 years) (*P* < 0.001). SRCC patients presented more frequently with stage III tumours than MC and NMC patients (79.4 vs. 57.1%, 52.8%, *P* < 0.001, respectively) and poorer differentiation (*P* < 0.001). Compared with RC patients in MC (40.9%) and NMC (35.1%) group, the patients in SRCC (51.6%) group were more likely to be assigned for PRT (*P* < 0.001).

### Prognostic factors in stage II and III RC patients

SRCC patients presented poorer 5 year survival than MC and NMC patients in both of stage II (43.60% vs. 66.28% and 73.10%, *P* < 0.001, respectively) and III RC (34.55% vs. 53.90% and 63.10%, *P* < 0.001, respectively). The result of multivariate survival analysis using Cox model concerning all stage II and III RC patients is showed in Table 2. From our analysis, the higher age (>65 years), the larger tumour size (>5 cm), and poorly differentiated tumour grade were all significant factors that worsened survival in stage II and III RC (*P* < 0.001, respectively). On the other hand, multiple tumour number and more number of lymph nodes examined (NO ≥ 12) were associated with better survival (*P* < 0.001, respectively). SRCC was an independent predictor of poor prognosis in stage III (HR, 1.985; 95% CI, 1.607–2.452; *P* < 0.001), but not in stage II patients (*P* = 0.136). Stage III patients benefited from PRT (HR, 0.799; 95% CI, 0.748–0.854; *P* < 0.001), while the stage II did not (*P* = 0.442).

### SRCC as a poor prognostic factor in stage II and III RC patients treated with PRT

**Figure 1:**
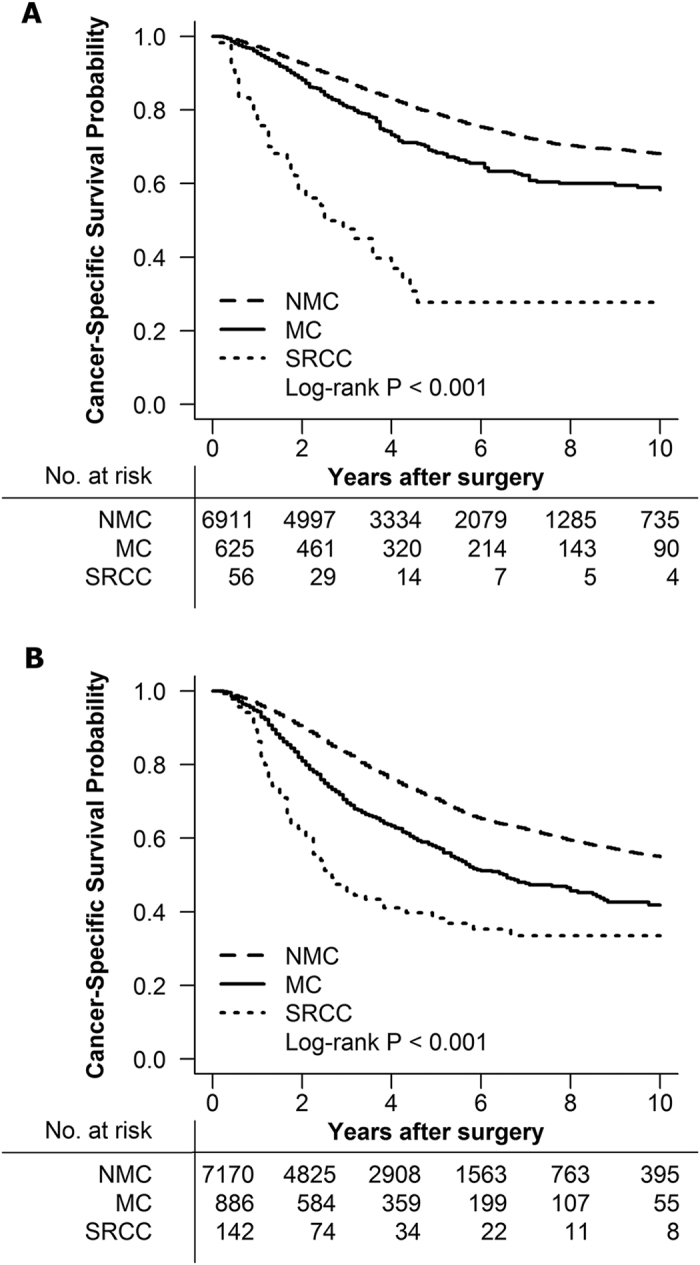
(**A**) Cancer specific survival for stage II rectal SRCC, MC and NMC patients underwent preoperative radiotherapy. (**B**) Cancer specific survival for stage III rectal SRCC, MC and NMC patients underwent preoperative radiotherapy.

There were 7,592 (48.1%) stage II patients and 8,198 (51.9%) stage III patients underwent the PRT. SRCC patients underwent PRT had a statistically significant worse cancer-specific survival (CSS) compared with MC and NMC patients in both stage II and III RC patients (Fig. 1A, B, log-rank, *P* < 0.001, respectively).

### PRT for stage II and III rectal SRCC patients

MC and NMC patients were divided into PRT plus surgery group and surgery alone group. As is shown in the Supplementary Fig. S1A, there was no statistically survival difference between the two group in stage II MC patients (log-rank, *P* = 0.394). But, PRT plus surgery group had a better CSS than that with surgery alone group in stage III MC patients (Supplementary Fig. S1B), stage II (Supplementary Fig. S2A) and III NMC patients (Supplementary Fig. S2B, log-rank, *P* < 0.001, respectively).

**Figure 2:**
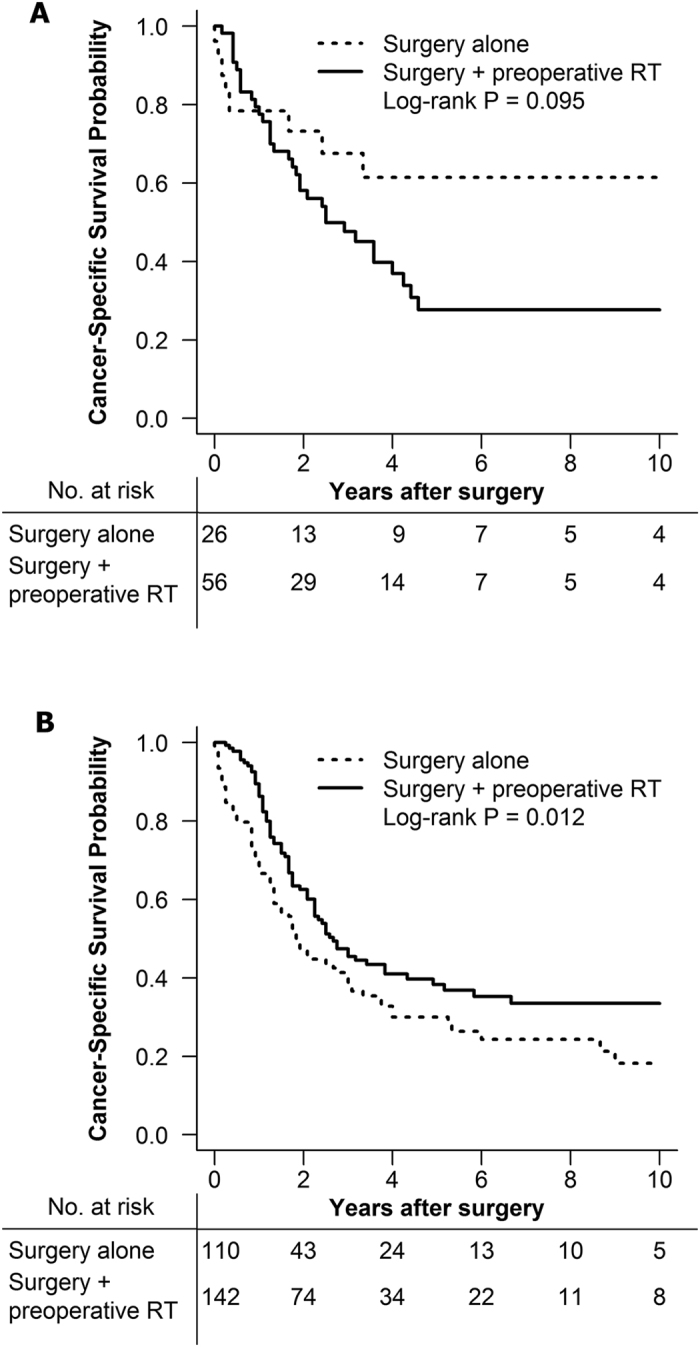
(**A**) Cancer specific survival for stage II rectal SRCC patients treated with or without preoperative radiotherapy. (**B**) Cancer specific survival for stage III rectal SRCC patients treated with or without preoperative radiotherapy. MC = mucinous adenocarcinoma; NMC = nonmucinous adenocarcinoma; SRCC = signet-ring cell carcinoma; RT = radiotherapy.

In 622 rectal SRCC patients underwent surgery, 210 patients received PRT. Excluding 12 patients without survival information, 198 patients (stage II, n = 56; stage III, n = 142) remained for analysis and were divided into PRT plus surgery group and surgery alone group. In stage II SRCC patients, there was no statistically survival difference between PRT plus surgery group and surgery alone group (Fig. 2A, log-rank, *P* = 0.095). Nevertheless, the stage III SRCC patients treated with PRT plus surgery had a better CSS than that with surgery alone (Fig. 2B, log-rank, *P* < 0.001). As Table 3 shown, PRT was not a significantly benefit factor in a total of stage II and stage III SRCC patients (HR, 0.911; 95% CI, 0.609–1.362; *P* = 0.648). The further multivariate survival analysis were stratified by each stage. It demonstrated PRT was an independent prognostic factor associated with better CSS in stage III SRCC patients (Table 4. HR, 0.611; 95% CI, 0.407–0.919; *P* = 0.018).

## Discussion

In this population based study, we analysed 69,543 locally advanced RC patients who were registered in the SEER. 0.9% of our population consisted of SRCC, similar to the numbers reported in previous literatures concerning on all stages CRC^5^,^9^,^10^,^11^. However, this proportion is much less than that of an Indian research, in which, SRCC comprised about 15.3 percent of stage II and III RC patients^4^. Many well-recognized SRCC associated features, such as younger diagnosis age, more frequently with advanced tumour, poorer differentiation were confirmed in this study^4^,^5^,^10^,^11^.

SRCC has been associated with poor prognosis compared with common adenocarcinoma^9^,^20^. Prognostic factors analysis stratified by tumour stage is rare in previous literatures. By a univariate analysis, Niek Hugen *et al*.^21^ showed a relatively worse survival for SRCC compared with non-SRCC in stage II and III CRC, prominently in stage III. We presented that SRCC was an independent predictor of poor prognosis in stage III but not in stage II RC, which had never been reported before. One of the reasons for the poor outcome in SRCC may be low differentiation at diagnosis, which was related to high risk of vascular invasion and lymph node involvement^4^,^22^. Ho-Su Lee and his colleagues^20^ found that most of the SRCC patients presented with an infiltrative growth pattern, which had been proved to be an independent prognostic factor among stage I-III CRC patients^13^. Moreover, Vallam *et al*.^4^ reported that circumferential resection margin (CRM) positivity rate was higher in SRCC (19%) than that in Non-SRCC (4%). The CRM positivity makes a curative surgery therapy impossible and leads to a high risk of local recurrence and poor survival^4^,^10^,^11^,^22^. The special metastatic pattern in SRCC may be another explanation for the adverse survival^12^,^23^. Signet ring cells usually present as single cell or gather as loose clusters, and disrupt cell-cell adhesion which contributes to the aggressive biological behavior. By breaking the E-cadherin/β-catenin complex and amplification of Bcl-2^24^, signet-ring cells can further reduce cell–cell adhesion, loosen the surrounding structure and spread far away. This may be one of explanations for why SRCCs tend to present peritoneal metastases. These metastases cannot be treated by radical surgery which lead directly to a poor prognosis. The genetic variations had been reported to affect survival of CRC^25^. Furthermore, these genetic factors including RAS (KRAS and NRAS), BRAF, MMR/MSI status maybe linked to chemoradiotherapy efficacy. We will investigate the relationship between genetic factors and chemoradiotherapy efficacy in SRCC in the future.

Due to the low frequency, SRCC has been rarely studied in a PRT setting. In the previous studies, there are probably biases associated with single-institution reporting and relatively small sizes^17^,^26^. To our best knowledge, this is the first study analysed outcome of PRT in rectal SRCC patients with long follow up information from multiple institutions. The present study demonstrated that CSS of SRCC patients was poorer than MC and NMC regardless of stages (both stage II and III) in a PRT setting. As mentioned above, SRCC presents a higher CRM positivity rate and infiltrative growth pattern, which mean higher post-operative residual tumour rates. Therefore, even treated with PRT, the SRCC had high recurrence rate and poor prognosis. Bratland *et al*.^17^ studied a cohort of 120 RC patients received PRT including six SRCC patients. It showed that rectal SRCC tend to have extensive mesorectal lymph node metastases and extramesorectal lymph node disease within the pelvic cavity which might be associated with poor PRT response. Some researchers tried to ascribe this poor PRT response to radiation resistance. Hugen N *et al*.^16^ found that mutated KRAS as a biomarker was strongly associated with poor response to chemoradiotherapy in MC. Though with a higher rate of microsatellite instability (MSI) in MC, the predictive value of MSI in response to radiotherapy is still controversial^27^,^28^,^29^. Expression of high-mobility group box 1, Paf15 and several microRNAs had been reported to be associated with response to chemoradiotherapy^30^,^31^,^32^,^33^,^34^. Unfortunately, neither lymph node dissemination nor molecular phenotype status had been recorded in our study and these analyses could not be performed. Further study is still needed to provide additional insight into the molecular mechanism underlying the pathogenesis of SRCC associated with radiation therapy response.

Some researchers thought these stage II patients with lower risk of local recurrence (clear margins and favorable prognosis features) maybe adequately treated with surgery and adjuvant chemotherapy. Many patients were under-staged by preoperative clinical imaging but subsequently proved to have positive lymph nodes in the surgical specimens. Therefore, the PRT in stage II RC remains controversial. Our research implied that stage II rectal SRCC patients did not receive benefit from PRT. The survival analysis revealed an even worse survival of this subgroup than patients who did not undergo PRT, although the difference was not statistically significant. Therefore, PRT may not have been necessary for these patients. The result should be interpreted caution because of the limited number of patients with stage II rectal SRCC in current study. In order to avoid side effects from unnecessary PRT for stage II RC, further study is needed to establish patients selected criterion to benefit this group. Interestingly, our study showed that PRT was associated with improved survival in stage III rectal SRCC. This was in accordance with the study of Bratland and his colleagues^17^. They suggested that patients with stage III rectal SRCC, when presenting limited lymph node metastasis, should be offered PRT in a tentatively curative setting. However, what worth mentioning is that PRT with improved survival may result in selected patient groups with significant tumour response and patients presenting with limited lymph node disease, similar views can be found in previous literatures^35^,^36^. We suggested that stage III rectal SRCC should be arranged for PRT. It could reduce the tumour volume and may facilitate the tumour resection, block the tumour invasion like lymph nodes metastasis or mesorectal fascia threatened, and reduce the local recurrence rate^37^.

Including the large number of patients from national population-based data, our study avoided the biases associated with single-institution experiences or limited sample sizes. Due to the nonrandomized nature of SEER, several limitations of current study deserved comment. Firstly, reviewing the individual pathological diagnosis was not feasible in a large population size. Variations in interpretation among pathologists may have led to misclassification. To explore the possible heterogeneity among each registration centre, we compared the proportion of SRCC, and there were no significant differences. Secondly, the SEER registry does not include detail information concerning the dose or duration of chemoradiation including PRT. Therefore, we were not able to take differences in PRT practice into account over the study period. Furthermore, some of the patients in our study may have underwent an emergency surgery due to bowel obstruction, thereby comprising a larger share in the patient group that did not receive PRT. Although data regarding cancer recurrences was not available in present study, cancer-specific survival is a reasonable surrogate of rectal cancer-specific outcome. Despite these, the results of the current research may provide some information for future studies of SRCC in relation to PRT in this area. In order to obtain a more definitive conclusion, further larger randomized controlled trial of Chinese population will be conducted through a multicentre cooperation.

In summary, our results showed that SRCC was a distinct entity that more often affected younger patients, presented more advanced tumour, poorer differentiation at diagnosis compared to non-signet ring cell carcinoma. SRCC remained a poor prognostic factor in locally advanced (stage II and III) RC patients who underwent PRT. Although PRT for stage II rectal SRCC was not associated with improved survival, it was an independent prognostic factor associated with better CSS in stage III SRCC patients. Further study is needed to elucidate rectal SRCC patients’ selection criterion for PRT.

## Patients and Methods

### SEER database

The data on all RC patients who underwent surgery between 1988 and 2011were retrieved from the Surveillance, Epidemiology, and End Results (SEER) database. The SEER database covered and published the information of cancer incidence and survival from 18 population-based cancer registries representing approximately 30% of the United States population (http://seer.cancer.gov/ about/overview.html). This version of SEER database we used had been released April 2014 (November 2013 submission). Characteristics recorded for each patient included age at diagnosis, gender, race, year of diagnosis, tumour numbers, tumour size, TNM stage, histological type, histological grade, surgery carried out and receipt of preoperative radiation therapy. Tumours were classified according to the International Classification of Diseases for Oncology (ICD-O). All TNM classification was restaged according to the criteria described in the American Joint Committee on Cancer (AJCC) Cancer Staging Manual, 7^th^ edition, 2010 (Stages I, II, III, and IV). The locally advanced RC patients (stage II and III) were included in this study. Based on the International Classification of Diseases for Oncology (third edition, ICD-O-3) coding schema, the tumour histological subtypes were identified as SRCC (8490), MC (8480, 8481) and NMC (8010, 8140–8141, 8144–8145, 8210–8211, 8220–8221, 8230–8231, 8260–8263). Histological grade was classified as well differentiated (G1), moderately differentiated (G2), poorly differentiated (G3), and undifferentiated (G4). The cancer-specific survival (CSS) time was calculated from the date of diagnosis to the date of cancer-specific death or the end of follow-up (cutoff date: December 2011). Deaths attributed to the cancer of interest (RC) were treated as events, and deaths from other causes are treated as censored observation.

### Ethics Statement

This study was based on data from the SEER database, which contain no identifiers and were publicly available. We obtained permission to access research data files with the reference number 10058-Nov2013, and this study was approved by the ethics committee of Sichuan University West China Hospital. Informed consent from patients was not required due to the study’s retrospective nature. The analysis did not involve interaction with human subjects or use personal identifying information. Patient records/information was anonymized and de-identified prior to analysis, and the methods were performed in accordance with the approved guidelines.

### Statistical Analysis

The R version 3.1.2 (http://www.R-project.org/) was used to perform all statistical analysis. The *t* test or chi-square test was used to compare clinicopathological characteristics. Survival curves were based on Kaplan–Meier method. The differences between the curves were analysed by log-rank test. Univariate and multivariate survival analyses were examined by the Cox proportional hazard models. The data were presented as hazard ratios (HR) with 95% confidence intervals (CI). All statistical tests were performed 2-sided, and *P* values <0.05 were considered to be statistically significant.

## Additional Information

**How to cite this article**: Ling, C.-R. *et al*. Prognosis and value of preoperative radiotherapy in locally advanced rectal signet-ring cell carcinoma. *Sci. Rep.* **7**, 45334; doi: 10.1038/srep45334 (2017).

**Publisher's note:** Springer Nature remains neutral with regard to jurisdictional claims in published maps and institutional affiliations.

## Supplementary Material

Supplementary Figures

## Figures and Tables

**Table 1:** Clinical and pathological characteristics of rectal SRCC, MC and NMC patients.

Characteristics	SRCC	MC	NMC	*P* value	*P* value	*P* value
	n = 622	n = 5,885	n = 63,036	SRCC vs MC	SRCC vs NMC	MC vs NMC
Age (years, Mean ± SD)	59.4 ± 17.0	65.4 ± 13.9	65.4 ± 14.3	<0.001	<0.001	0.002
Gender						
Female	217 (34.9)	2,469 (42.0)	36,271 (57.5)	0.001	<0.001	0.453
Male	405 (65.1)	3,416 (58.0)	26,765 (42.5)			
Race						
White	496 (79.9)	4,925 (83.8)	52,203 (82.9)	<0.001	0.066	<0.001
Black	50 (8.0)	525 (8.9)	4,825 (7.7)			
Others	75 (12.1)	430 (7.3)	5,924 (9.4)			
Unknown	1	5	84			
Year of Diagnosis						
1988–2000	200 (32.2)	2,294 (39.0)	22,975 (36.4)	0.001	0.027	<0.001
2001–2011	422 (67.8)	3,591 (61.0)	40,061 (63.6)			
Tumour numbers						
Single	480 (77.2)	4,243 (72.1)	47,083 (74.7)	0.007	0.157	<0.001
Multiple	142 (22.8)	1,642 (27.9)	15,951 (25.3)			
Unknown	0	0	2			
Tumour size (cm)						
≤5	277 (54.7)	2,875 (56.8)	37,834 (68.9)	0.372	<0.001	<0.001
>5	229 (45.3)	2,186 (43.2)	17,098 (31.1)			
Unknown	116	824	8,104			
TNM stage						
II	128 (20.6)	2,523 (42.9)	29,750 (47.2)	<0.001	<0.001	<0.001
III	494 (79.4)	3,362 (57.1)	33,286 (52.8)			
Tumour grade						
Well	8 (1.5)	524 (10.0)	3,752 (6.2)	<0.001	<0.001	<0.001
Moderately	36 (6.7)	3,452 (66.1)	46,070 (76.5)			
Poorly	453 (84.0)	1,154 (22.1)	9,949 (16.5)			
Undifferentiated	42 (7.8)	90 (1.7)	455 (0.8)			
Unknown	83	665	2,810			
NO. of lymph nodes examined						
<12	307 (51.7)	3,131 (54.8)	32,269 (52.5)	0.151	0.705	0.001
≥12	287 (48.3)	2,586 (45.2)	29,240 (47.5)			
Unknown	28	168	1,527			
Preoperative Radiotherapy						
No	197 (48.4)	2,423 (59.1)	28,737 (64.9)	<0.001	<0.001	<0.001
Yes	210 (51.6)	1,676 (40.9)	15,558 (35.1)			
Unknown	215	1,786	18,741			

**Table 2:** Multivariate Cox proportional hazards model predicting death in stage II and III rectal cancer.

Variable	Stage II			Stage III		
	HR	95% CI	*P* value	HR	95% CI	*P* value
Gender						
Female	1.000			1.000		
Male	1.026	0.961 to 1.096	0.442	1.098	1.041 to 1.159	0.001
Age (years)						
≤mean 65	1.000			1.000		
>mean 65	1.785	1.660 to 1.918	<0.001	1.749	1.654 to 1.849	<0.001
Race						
White	1.000			1.000		
Black	1.409	1.260 to 1.575	<0.001	1.320	1.205 to 1.445	<0.001
Others	0.807	0.712 to 0.913	0.001	0.890	0.813 to 0.974	0.011
Year At Diagnosis						
1988–2000	1.000			1.000		
2001–2011	0.962	0.573 to 1.616	0.431	0.766	0.723 to 0.811	<0.001
Tumour numbers						
Single	1.000			1.000		
Multiple	0.645	0.586 to 0.711	<0.001	0.618	0.566 to 0.675	<0.001
Tumour size (cm)						
≤5	1.000			1.000		
>5	1.313	1.227 to 1.405	<0.001	1.282	1.211 to 1.358	<0.001
Histological Type						
NMC	1.000			1.000		
MC	1.248	1.110 to 1.403	<0.001	1.304	1.193 to 1.425	<0.001
SRCC	1.544	0.873 to 2.731	0.136	1.985	1.607 to 2.452	<0.001
Tumour grade						
Well	1.000			1.000		
Moderately	1.151	1.013 to 1.306	0.030	1.093	0.968 to 1.235	0.152
Poorly	1.427	1.229 to 1.657	<0.001	1.606	1.412 to 1.827	<0.001
Undifferentiated	1.305	0.775 to 2.197	0.316	2.080	1.602 to 2.701	<0.001
NO. of lymph nodes examined						
<12	1.000			1.000		
≥12	0.669	0.622 to 0.720	<0.001	0.919	0.870 to 0.970	0.002
Preoperative Radiotherapy						
No	1.000			1.000		
Yes	0.968	0.892 to 1.051	0.442	0.799	0.748 to 0.854	<0.001

**Table 3:** Multivariate survival analysis using Cox model concerning rectal cancer SRCC patients with stage III.

Variable	SRCC		
	HR	95% CI	*P* value
Gender			
Female	1.000		
Male	0.876	0.579 to 1.325	0.529
Age (years)			
≤mean 65	1.000		
>mean 65	2.052	1.283 to 3.283	0.003
Race			
White	1.000		
Black	2.920	1.355 to 6.295	0.006
Others	1.036	0.598 to 1.794	0.900
Year At Diagnosis			
1988–2000	1.000		
2001–2011	0.825	0.514 to 1.324	0.425
Tumour numbers			
Single	1.000		
Multiple	0.655	0.323 to 1.328	0.241
Tumour size (cm)			
≤5	1.000		
>5	1.869	1.244 to 2.810	0.003
NO. of lymph nodes examined			
<12	1.000		
≥12	1.216	0.771 to 1.917	0.401
Preoperative Radiotherapy			
No	1.000		
Yes	0.611	0.407 to 0.919	0.018

**Table 4:** Multivariate survival analysis using Cox model concerning rectal cancer patients with SRCC.

Variable	SRCC		
	HR	95% CI	*P* value
Gender			
Female	1.000		
Male	0.745	0.486 to 1.141	0.175
Age (years)			
≤mean 65	1.000		
>mean 65	2.137	1.346 to 3.393	0.001
Race			
White	1.000		
Black	1.859	0.704 to 4.909	0.211
Others	1.041	0.606 to 1.789	0.883
Year At Diagnosis			
1988–2000	1.000		
2001–2011	0.962	0.573 to 1.616	0.885
Tumour numbers			
Single	1.000		
Multiple	0.634	0.313 to 1.284	0.206
Tumour size (cm)			
≤5	1.000		
>5	1.753	1.163 to 2.643	0.007
TNM stage			
II	1.000		
III	2.252	1.166 to 4.347	0.016
Tumour grade			
Well	1.000		
Moderately	6645.710	0 to 1.510 × 10^71^	0.911
Poorly	5148.098	0 to 1.168 × 10^71^	0.914
Undifferentiated	6795.919	0 to 1.544 × 10^71^	0.911
NO. of lymph nodes examined			
<12	1.000		
≥12	1.218	0.786 to 1.888	0.378
Preoperative Radiotherapy			
No	1.000		
Yes	0.911	0.609 to 1.362	0.648
