# Supplementary material for: Prognosis and value of preoperative radiotherapy in locally advanced rectal signet-ring cell carcinoma
Source: Sci Rep. 2017 Mar 27;7:45334. doi: 10.1038/srep45334 (PMC5366911; doi:10.1038/srep45334)
Supplement: Supplementary Figures [file srep45334-s1.pdf]

Prognosis and value of preoperative radiotherapy in locally advanced rectal signet-ring cell carcinoma

Authors:

Chun-Run Ling[1,+], MM; Rui Wang[2,+], MD PhD; Mo-Jin Wang[1,+], MD PhD; Jie Ping[3,*], PhD; Wen Zhuang[1,*], MD PhD

1 Department of Gastrointestinal Surgery, West China Hospital, Sichuan University, Chengdu 610041, China

2 Department of Gastroenterology, West China Hospital, Sichuan University, Chengdu 610041, China

3 Center for Quantitative Sciences, Vanderbilt University School of Medicine, Nashville, TN 37232, USA

+Chun-Run Ling, Rui Wang and Mo-Jin Wang share co-first authorship.

***Corresponding authors:**

Jie Ping, PhD. Center for Quantitative Sciences, Vanderbilt University School of Medicine, Nashville, TN 37232, USA

Fax: +1-6159362602 Email: jie.ping@vanderbilt.edu

Wen Zhuang, MD. PhD. Department of Gastrointestinal Surgery, West China Hospital, Sichuan University, Chengdu, China

37 Guo Xue Xiang, Chengdu 610041, Sichuan Province, China.

Fax: +86-02885422876 Email: zhuangwen1966@163.com

Supplementary Figures

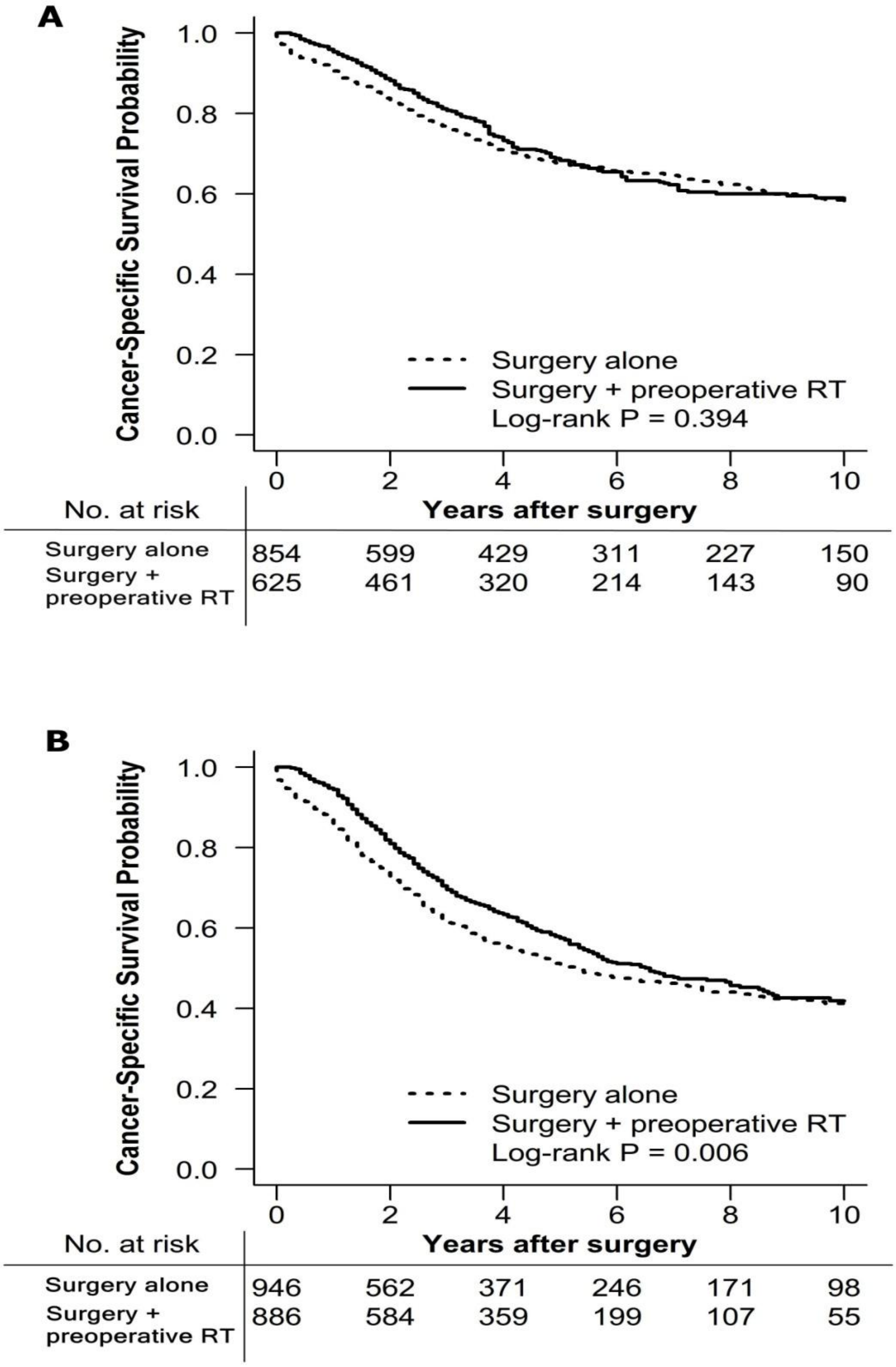

Supplementary Figure S1. (A) Cancer specific survival for stage II rectal MC patients treated with or without preoperative radiotherapy. (B) Cancer specific survival for stage III rectal MC patients treated with or without preoperative radiotherapy.

MC = mucinous adenocarcinoma; RT = radiotherapy.

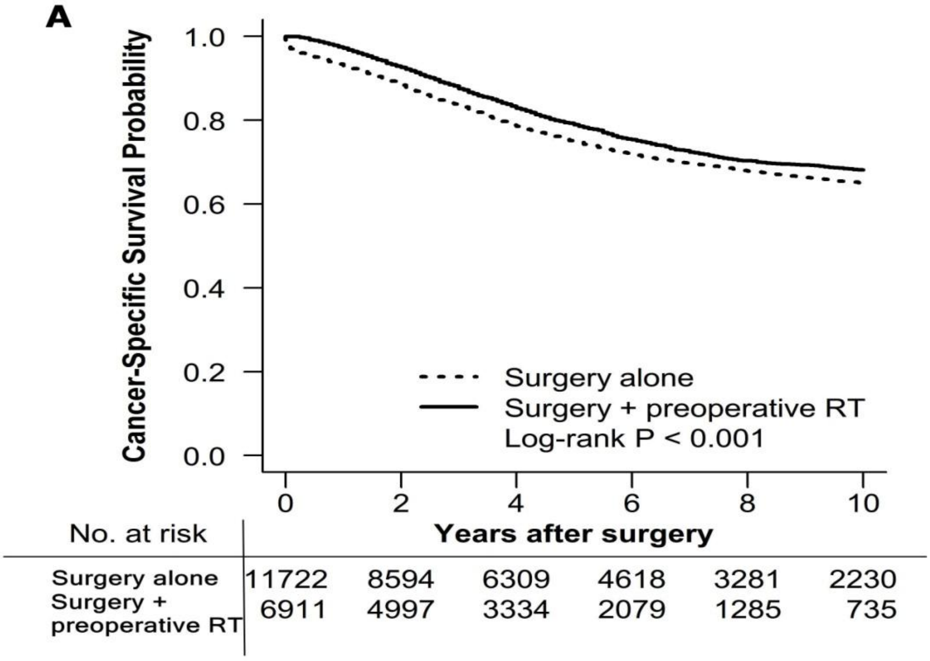

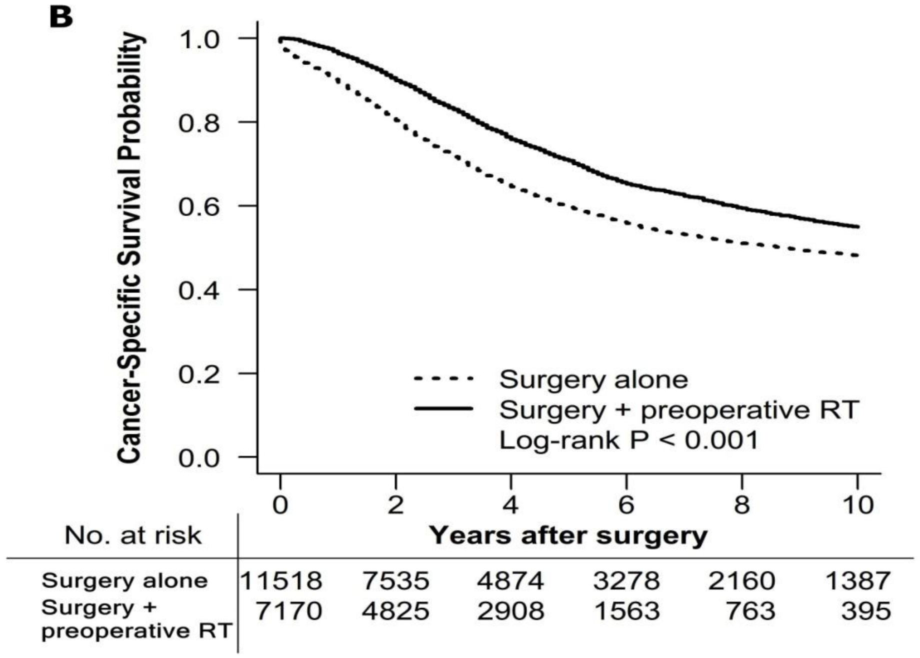

Supplementary Figure S2. (A) Cancer specific survival for stage II rectal NMC patients treated with or without preoperative radiotherapy. (B) Cancer specific survival for stage III rectal NMC patients treated with or without preoperative radiotherapy.

NMC = nonmucinous adenocarcinoma; RT = radiotherapy.
